# Report from the CVOT Summit 2021: new cardiovascular, renal, and glycemic outcomes

**DOI:** 10.1186/s12933-022-01481-0

**Published:** 2022-04-08

**Authors:** Oliver Schnell, Tadej Battelino, Richard Bergenstal, Matthias Blüher, Michael Böhm, Frank Brosius, Richard D. Carr, Antonio Ceriello, Thomas Forst, Francesco Giorgino, Bruno Guerci, Hiddo J. L. Heerspink, Baruch Itzhak, Linong Ji, Mikhail Kosiborod, Nebojša Lalić, Michael Lehrke, Nikolaus Marx, Michael Nauck, Helena W. Rodbard, Giuseppe M. C. Rosano, Peter Rossing, Lars Rydén, Francesca Santilli, Petra-Maria Schumm-Draeger, Per Olav Vandvik, Tina Vilsbøll, Christoph Wanner, Carol Wysham, Eberhard Standl

**Affiliations:** 1 Forschergruppe Diabetes e. V., Helmholtz Center Munich, Ingolstaedter Landstraße 1, 85764 Munich, Germany; 2 University Medical Center, Ljubljana, Slovenia; 3 University Children’s Hospital, Ljubljana, Slovenia; 4 Faculty of Medicine, University of Ljubljana, Ljubljana, Slovenia; 5 International Diabetes Center at Park Nicollet, Health Partners, Minneapolis, MN USA; 6 Department of Medicine, University of Leipzig, Leipzig, Germany; 7 Klinik für Innere Medizin III, Universitätsklinikum des Saarlandes, Saarland University, Homburg, Germany; 8 College of Medicine, University of Arizona, Tuscon, AZ USA; 9 University College London, London, UK; 10 IRCCS MultiMedica, Milan, Italy; 11 CRS Clinical Research Services Mannheim GmbH, Mannheim, Germany; 12 Department of Emergency and Organ Transplantation, University of Bari Aldo Moro, Bari, Italy; 13 Department of Endocrinology Diabetology and Nutrition, Nancy University Hospital, Nancy, France; 14 Faculty of Medicine, University of Lorraine, Vandoeuvre-lès-Nancy, France; 15 Department of Clinical Pharmacy and Pharmacology, University Medical Center Groningen, University of Groningen, Groningen, The Netherlands; 16 Clalit Health Services and Technion Faculty of Medicine, Haifa, Israel; 17 Peking University People’s Hospital, Xicheng District, Beijing, China; 18 Cardiometabolic Center of Excellence, University of Missouri-Kansas City, Kansas City, MO USA; 19 Faculty of Medicine, Clinic for Endocrinology, Diabetes and Metabolic Diseases, University Clinical Center of Serbia, University of Belgrade, Belgrade, Serbia; 20 Department of Internal Medicine I, University Hospital Aachen, Aachen, Germany; 21 Diabetes Division, Katholisches Klinikum Bochum, St. Josef-Hospital, Ruhr University Bochum, Bochum, Germany; 22 Endocrine and Metabolic Consultants, Rockville, MD USA; 23 Department of Medical Sciences, IRCCS, Rome, Italy; 24 Steno Diabetes Center Copenhagen, Herlev, Denmark; 25 Department of Clinical Medicine, University of Copenhagen, Copenhagen, Denmark; 26 Department of Medicine K2, Karolinska Institute, Stockholm, Sweden; 27 Department of Medicine and Aging, Hospital and, University of Chieti, Chieti, Italy; 28 Centre Internal Medicine Fünf Höfe, Munich, Germany; 29 Department of Medicine, Lovisenberg Diaconal Hospital, Oslo, Norway; 30 Center for Diabetes Research, Gentofte Hospital, University of Copenhagen, Hellerupn, Denmark; 31 University Hospital of Würzburg, Würzburg, Germany; 32 Section of Endocrinology and Metabolism, MultiCare Rockwood Clinic, Spokane, WA USA

**Keywords:** Diabetes, Cardiovascular disease, Heart failure, Chronic kidney disease, Obesity, SGLT2 inhibitor, GLP-1 receptor agonist, Tirzepatide, Mineralocorticoid receptor antagonist, Living guidelines

## Abstract

The 7th Cardiovascular Outcome Trial (CVOT) Summit on Cardiovascular, Renal, and Glycemic Outcomes, was held virtually on November 18–19, 2021. Pursuing the tradition of the previous summits, this reference congress served as a platform for in-depth discussion and exchange on recently completed CVOTs. This year’s focus was placed on the outcomes of EMPEROR-Preserved, FIGARO-DKD, AMPLITUDE-O, SURPASS 1–5, and STEP 1–5. Trial implications for diabetes and obesity management and the impact on new treatment algorithms were highlighted for endocrinologists, diabetologists, cardiologists, nephrologists, and general practitioners. Discussions evolved from outcome trials using SGLT2 inhibitors as therapy for heart failure, to CVOTs with nonsteroidal mineralocorticoid receptor antagonists and GLP-1 receptor agonists. Furthermore, trials for glycemic and overweight/obesity management, challenges in diabetes management in COVID-19, and novel guidelines and treatment strategies were discussed.

*Trial registration* The 8th Cardiovascular Outcome Trial Summit will be held virtually on November 10–11, 2022 (http://www.cvot.org)

## Background

Diabetes mellitus is on the rise across the globe. Prevalence estimates in the 20–79-year age group have increased from 463 million (9.3% of the world population) in 2019 to 537 million (10.5%) in 2021 [1, 2]. According to the World Health Organization (WHO), 44% of people with diabetes have overweight or obesity [3]. The International Diabetes Federation (IDF) predicts that the diabetes prevalence will rise to 783.2 million by 2045 (12.2%), with a relatively mild increase in the proportion of people with diabetes of 13% in Europe, 24% in North America and the Caribbean, and 27% in the Western Pacific [1, 2]. Higher increases will be noticeable in South and Central America (50%), South-East Asia (68%), and the Middle East and North Africa (87%). The highest increase is predicted for Sub-Saharan Africa (134%) [1]. The increasing diabetes prevalence is accompanied by a rise in direct diabetes costs. According to the IDF, the total estimated global healthcare expenditure for people with diabetes aged 20–79 increased from 232 billion USD in 2007 to 966 billion USD in 2021 [1, 2].

Diabetes is in the long-term commonly accompanied by at least one comorbidity. Nearly 75% of persons with diabetes have concomitant hypertension [4]. Cardiovascular disease (CVD) is a major comorbidity of diabetes [5, 6]. A metanalysis including more than 4.5 million persons with type 2 diabetes mellitus (T2D) indicated that 32.2% of the subjects had CVD, including 29.1% with atherosclerosis, 21.2% with coronary heart disease, 14.9% with heart failure (FH), 14.6% with angina, 10.0% with myocardial infarction (MI), and 7.6% with stroke [5]. Cardiovascular (CV) deaths comprised 50.3% of all deaths [5]. Furthermore, long-term elevated glucose levels cause damage to the nervous system. In this regard, 13–26% of people with diabetes have a chronic painful distal symmetric sensorimotor polyneuropathy [7, 8]. The kidney is another organ affected by diabetes. It has been shown that approximately 20–40% of patients with diabetes develop kidney disease due to diabetes (DKD) [9, 10]. In persons with T2D and diagnosed DKD, life expectancy is estimated to be reduced by 16 years [11].

Because of the health-compromising comorbidities of diabetes and the quality-of-life impairment of affected individuals, the continuous development of effective, accessible, affordable, and safe medications is necessary.

Regarding drugs’ safety, the U.S. Food and Drug Administration (FDA) in 2008 issued a guidance to evaluate CV risk in new glucose lowering therapies for T2D as a response to the potentially elevated risk for micro- and macrovascular events of some glucose-lowering medications [12, 13]. Since then, cardiovascular outcome trials (CVOTs) have been conducted, mainly for three glucose-lowering medication classes: glucagon-like peptide-1 receptor agonists (GLP-1 RAs), dipeptidylpeptidase-4 inhibitors (DPP-4is), and sodium–glucose cotransporter-2 inhibitors (SGLT2is). By 2020, five CVOTs have been conducted for DPP-4is [14–18]. For the SGLT2is, five CVOTs [19–23], two kidney outcome trials [24, 25], and three HF outcome trials [26–28] were published. Regarding the GLP-1 RAs, seven CVOTs have been conducted [29–33]. In addition, a renal outcome trial for a novel mineralocorticoid receptor antagonist (MRA) in patients with chronic kidney disease (CKD) and T2D was published [34]. In 2021, the list of outcome trials was expanded by two further CVOTs (AMPLITUDE-O—Efpeglenatide [35] and FIGARO-DKD—Finerenone [36]) and a HF outcome trial in patients with HF and a preserved ejection fraction (HFpEF) with or without diabetes (EMPEROR-Preserved—Empagliflozin [37]).

In addition to CVOTs, five global trials of the SURPASS program (SURPASS 1–5) investigating the efficacy and safety of tirzepatide, a novel dual glucose-dependent insulinotropic polypeptide (GIP) and glucagon-like peptide-1 (GLP-1) receptor agonist, with regard to its glucose-lowering effect in persons with T2D were published [38–42]. Furthermore, the results of four trials of the STEP clinical trial program (STEP 1–5) evaluating the effect of the GLP-1 RA semaglutide 2.4 mg on weight reduction in persons with or without T2D were issued [43–46].

As in previous years [47–52], we present and summarize key aspects discussed at the seventh CVOT Summit held virtually on 18–19 November 2021. The CVOT Summit on Cardiovascular, Renal, and Glycemic Outcomes 2021, was an interdisciplinary platform, which was also organized in conjunction with four study groups: Primary Care Diabetes Europe (PCDE, www.pcdeurope.org), European Diabetic Nephropathy Study Group (EDNSG, www.ednsg.org), the Incretin Study Group (www.easd-incretin.ku.dk), and the Working Group Diabetes & Herz (www.ddg.org). Participants from 88 countries and five continents with specialties in diabetology, endocrinology, cardiology, nephrology, and primary care contributed to the discussions of the CVOT Summit on Cardiovascular and Renal Outcomes 2021 (www.cvot.org).

## Updates on CVOTs

A summary of the characteristics and results of HF and CV outcome trials published in 2021 is listed in Tables 1, 2 and 3.

**Table 1:** Overview of basic characteristics of heart failure and cardiovascular outcome trials published in 2021

Study name	Study status	Drug	Drug class	Intervention	Primary outcome	n	Median follow up	Start and end date	Clinicaltrials.gov ID
EMPEROR-preserved [37]	Completed	Empagliflozin	SGLT2 inhibitor	Empagliflozin 10 mg once daily vs. placebo	Composite of CV death or HHF	5988	2.2	03.2017–04.2021	NCT03057951
FIGARO-DKD [36]	Completed	Finerenone	Mineralocorticoid receptor antagonist	Finerenone 10 mg or 20 mg once daily vs. placebo	Composite of death from CV causes, nonfatal MI, nonfatal stroke, or HHF	8246	3.4	09.2015–02.2021	NCT02545049
AMPLITUDE-O [35]	Terminated	Efpeglenatide	GLP-1 receptor agonist	Efpeglenatide 4 mg or 6 mg subcutaneous once a week vs. placebo	Composite of nonfatal MI, nonfatal stroke, or death from CV or undetermined causes	4077	1.8	04.2018–12.2020	NCT03496298

*HHF* hospitalization for heart failure

**Table 2:** Heart failure outcome trials completed in 2021: comparison of active vs. placebo group

EMPEROR-preserved [37]		
Class and cardiovascular outcomes	HR (95% CI)	p-value
**Primary composite outcome**		
Composite of cardiovascular death or hospitalization for heart failure	0.79 (0.69–0.90)	< 0.001
**Secondary outcome**		
Total number of hospitalizations for heart failure	0.73 (0.61–0.88)	< 0.001
**Secondary outcome**		
Mean slope of change in eGFR per year—ml/min/1.73 m^2^	1.36 (1.06–1.66)	< 0.001
**Other prespecified analyses**		
Change in KCCQ clinical summary score at week 52	1.32 (0.45–2.19)	
**Other prespecified analyses**		
Total number of hospitalizations for any cause	0.93 (0.85–1.01)	
**Other prespecified analyses**		
Composite renal outcome	0.95 (0.73–1.24)	
**Other prespecified analyses**		
Onset of new diabetes in patients with prediabetes	0.84 (0.65–1.07)	
**Other prespecified analyses**		
Death from any cause	1.00 (0.87–1.15)	

Adverse events	Event rate (%) active vs. placebo group	
Urinary tract infections	9.9 vs. 8.1	
Genital infections	2.2 vs. 0.7	
Hypotension	10.4 vs. 8.6	

*KCCQ* Kansas City Cardiomyopathy Questionnaire

**Table 3:** Cardiovascular trials in diabetes completed in 2021: comparison of active vs. placebo group

FIGARO-DKD [36]—Finerenone		AMPLITUDE-O [35]—Efpeglenatide	
Class and cardiovascular outcomes	HR (95% CI), p-value	Class and cardiovascular outcomes	HR (95% CI), p-value
Number of participants	7437	Number of participants	4076
**Primary composite outcome**		**Primary composite outcome**	
Composite of CV death, nonfatal MI, nonfatal stroke, and HHF	0.87 (0.76–0.98), p = 0.03	Incident MACE	0.73 (0.58–0.92), ^+^p < 0.001
**Secondary outcome**		**Secondary outcome**	
Composite of onset of kidney failure, sustained ≥ 40% eGFR decline or death from renal causes	0.87 (0.76–1.01)	Expanded MACE composite outcome event	0.79 (0.65–0.96), *p = 0.02
**Secondary outcome**		**Secondary outcome**	
Hospitalization for any cause	0.97 (0.90–1.04)	Composite renal outcome event	0.68 (0.57–0.79), *p < 0.001
**Secondary outcome**			
All-cause mortality	0.89 (0.77–1.04)		
**Secondary outcome**			
Kidney composite outcome	0.77 (0.60–0.99)		

Adverse events	Event rate (%) active vs. placebo group	Adverse events	Event rate (%) active vs. placebo group (p-value)
Hyperkalemia	10.8 vs. 5.3	Severe gastrointestinal event	3.3 vs. 1.8 (p=0.009)
Hypokalemia	1.1 vs. 2.4		
Gynecomastia	0.1 vs. 0.1		

^+^p-value for noninferiority

^*^p-value for superiority

## SGLT2 inhibitors

### EMPEROR-Preserved (Table 2: HF outcome)

The EMPEROR-preserved trial [37] assessed the effect of empagliflozin (10 mg/daily) in 5988 patients, men and women, aged ≥ 18 years, with chronic HF (New York Heart Association (NYHA) class II, III or IV) for ≥ 3 months and an ejection fraction of more than 40% (HFpEF) [53]. The median duration of follow-up was 2.2 years. Nearly half of the patients had T2D (48.9% in the empagliflozin-treated group and 49.2% in the placebo group). The primary endpoint was a composite of CV death or hospitalization for heart failure (HHF). Regarding the secondary endpoints, the first one was the occurrence of HHF and the second one was the rate of decline in the estimated glomerular filtration rate (eGFR) during double blind treatment. Additional prespecified outcomes are presented in Table 2.

Empagliflozin showed a significant improvement of the primary composite outcome with a reduced combined risk for CV death or HHF (hazard ratio (HR) 0.79 [95% confidence interval (CI) 0.69–0.90]; p < 0.001). This result was mainly related to a lower risk of HHF (HR 0.71 [95% CI 0.60–0.83]) and a slight decrease in CV death risk (HR 0.91 [95% CI 0.76–1.09]). Subgroup analysis showed consistent benefit of empagliflozin on the primary composite outcome in patients with diabetes (HR 0.79 [95% CI 0.67–0.94]) or without diabetes (HR 0.78 [95% CI 0.64–0.95]). Regarding the first secondary outcome, a significant 27% relative reduction in the total number of HHF was reported (HR 0.79 [95% CI 0.69–0.90]). In addition, the decline rate in the eGFR (second secondary outcome) was slower in the empagliflozin-treated group compared to placebo (− 1.25 vs. − 2.62 ml/min/1.73 m^2^ per year; Between-group difference in slope: 1.36 ml/min/1.73 m^2^ per year [95% CI 1.06–1.66]; p < 0.001). The results of other prespecified analyses are presented in Table 2. Regarding adverse events, although in general infrequent, urinary tract infections, genital infections, and hypotension were somewhat more often with empagliflozin (Table 2) [37].

## GLP-1 receptor agonists

### AMPLITUDE-O (Table 3: CV outcome)

The randomized, placebo-controlled trial AMPLITUDE-O analyzed the effect of the exendin-based GLP-1 RA, efpeglenatide, on adverse CV events [35] in persons with T2D. The participants were 18 years or older, had glycated hemoglobin A1c (HbA1c) > 7%, a history of CVD or were ≥ 50 years old (if male) or ≥ 55 (if female) and had kidney disease [35]. 2717 of the 4076 participants received efpeglenatide. They were divided into two groups: The first group received efpeglenatide at a weekly dose of 2 mg for 4 weeks. The dose was then increased to 4 mg for the remaining duration of the study. The second group was treated with efpeglenatide 2 mg for 4 weeks, then 4 mg weekly for 4 weeks, and finally 6 mg weekly until the end of the study [35].

The primary composite outcome was the first occurrence of a major adverse CV event (MACE), defined as a composite of nonfatal MI, nonfatal stroke, or death from CV or undetermined causes. In addition, there were two key secondary outcomes: an expanded MACE (MACE, coronary revascularization, or hospitalization for unstable angina) and a composite renal outcome (incident macroalbuminuria, plus an increase in the urine albumin-to-creatinine ratio (UACR) of ≥ 30% from baseline, a continual decrease in the eGFR of ≥ 40% for ≥ 30 days, renal-replacement therapy for ≥ 90 days, or a continuous eGFR of < 15 ml/min/1.73m^2^ for ≥ 30 days) [35].

During a median follow-up of 1.81 years, efpeglenatide significantly reduced the relative risk of the primary composite outcome (MACE) by 27% (HR 0.73 [95% CI 0.58–0.92]; p < 0.001 for noninferiority). With regard to the key secondary outcomes, efpeglenatide showed a significant reduction in the incidence of at least one expanded MACE composite event (HR 0.79 [95% CI 0.65–0.96]; p = 0.02 for superiority) and at least one composite renal outcome event (HR 0.68 [95% CI 0.57–0.79]; p < 0.001 for superiority) [35].

Severe gastrointestinal events occurred significantly more often in the group assigned to receive efpeglenatide (3.3% vs. 1.8% for placebo; P = 0.009). They were mainly due to diarrhea, constipation, nausea, vomiting, or bloating (2.2% vs. 1.4% for placebo; P = 0.03) [35]. On the sponsor’s decision that was not related to safety concerns, the AMPLITUDE-O trial was terminated.

## Mineralocorticoid receptor antagonists

### FIGARO-DKD (Table 3: CV outcome)

The FIGARO-DKD trial assessed the cardiovascular and renal effects of the selective nonsteroidal mineralocorticoid receptor antagonist, finerenone, in patients with T2D and a wide range of CKD [36]. Eligible patients (4076 participants; ≥ 18 years old) had to have a UACR of 30–300 mg/g and an eGFR of 25–90 ml/min/1.73m^2^ (stage 2–4 CKD) or a UACR of 300–5000 mg/g and an eGFR ≥ 60 ml/min/1.73m^2^ (stage 1 or 2 CKD) [36]. All patients were treated with renin-angiotensin system (RAS) blockade at the maximum tolerated dose. The primary outcome was composite of death from CV causes, nonfatal MI, nonfatal stroke, or HHF. The key secondary outcome was a composite of the first occurrence of kidney failure, a sustained decrease from baseline of at least 40% in the eGFR for a period of at least 4 weeks, or death from renal causes. Main further secondary outcomes were hospitalization for any cause, death from any cause, and a kidney composite outcome (first onset of kidney failure, a sustained decrease from baseline of at least 57% in the eGFR for at least 4 weeks, or death from renal causes) [36].

In the finerenone-treated group, a significant decrease in the relative risk of the primary composite outcome by 13% was observed (HR 0.87 [95% CI 0.76–0.98]; p = 0.03). The effect was primarily driven by a lower incidence of HHF (HR 0.71 [95% CI 0.56–0.90]). There was no significant decrease in the risk of the secondary composite outcome (occurrence in 9.5% in the finerenone group (N = 3686) and 10.8% in the placebo group (N = 3666); HR 0.87 [95% CI 0.76–1.01]). Also, no significant reduction in the risk for the secondary outcomes (hospitalization for any cause and death from any cause) was observable. The kidney secondary composite outcome (first onset of kidney failure, a sustained decrease from baseline of at least 57% in the eGFR for at least 4 weeks, or death from renal causes) occurred in 2.9% in the finerenone group and 3.8% in the placebo group [36].

The hyperkalemia incidence was twofold higher with finerenone (10.8%) than placebo (5.3%). Also, the incidence of permanent discontinuation of the trial regimen due to hyperkalemia was higher with finerenone than with placebo (1.2% vs. 0.4%). Furthermore, patients treated with finerenone showed higher mean serum potassium levels (> 5.5 mmol/l) than patients who received a placebo (13.5% vs. 6.4%). The hypokalemia incidence was lower in the finerenone-treated group than in the placebo group (1.1% vs. 2.4%). There was no difference in the gynecomastia incidence between finerenone and placebo (0.1% vs. 0.1%). Two phase 3 trials investigating the efficacy and safety of finerenone in patients with HFpEF (FINEARTS-HF) and in patients with non-diabetes-related CKD (FIND-CKD) are at present being conducted [54, 55].

## Glycemic outcome trials

### SURPASS trials: (Tables 4 and 5)

**Table 4:** Key information of the SURPASS 1–6 clinical trials with the glucose-lowering dual GIP/GLP-1 receptor agonist tirzepatide

Study name	Clinicaltrials.gov ID	Study status	n	Key inclusion criteria	Key exclusion criteria	Intervention	Adjunctive therapy
SURPASS-1 [38]	NCT03954834	Completed	478	Naive to diabetes injectable therapies, HbA1c 7.0–9.5%, BMI ≥ 23 kg/m^2^ at screening	Use of any oral antihyperglycemic medications for 3 months before screening, eGFR < 30 ml/min/1.73m^2^	Tirzepatide 5, 10, or 15 mg SC once a week vs. placebo	None
SURPASS-2 [39]	NCT03987919	Completed	1879	HbA1c 7.0–10.5%, BMI ≥ 25 kg/m^2^ at screening Stable background medications (metformin)	eGFR < 45 ml/min/1.73m^2^	Tirzepatide 5, 10, or 15 mg SC once a week vs. semaglutide 1 mg once a week	Metformin
SURPASS-3 [40]	NCT03882970	Completed	1444	HbA1c 7.0–10.5%, BMI ≥ 25 kg/m^2^ at screening Stable background medications (metformin ± SGLT2i)	eGFR < 45 ml/min/1.73m^2^ Other medications than metformin ± SGLT2i	Tirzepatide 5, 10, or 15 mg SC once a week vs. insulin degludec SC once a day	Metformin or metformin + SGLT2i
SURPASS-4 [41]	NCT03730662	Completed	2002	HbA1c 7.0–10.5%, BMI ≥ 25 kg/m^2^ at screening Increased risk of CV events Stable background medications (metformin ± sulfonylurea or SGLT2i)	Other medications than metformin ± sulfonylurea or SGLT2i	Tirzepatide 5, 10, or 15 mg SC once a week vs. insulin glargine SC once a day	Metformin or metformin + sulfonylurea or SGLT2i
SURPASS-5 [42]	NCT04039503	Completed	457	HbA1c 7.0–10.5%, BMI ≥ 23 kg/m^2^ at screening Stable background medications (insulin glargine (U100) ± metformin)	eGFR < 30 ml/min/1.73m^2^ (< 45 if treated with metformin)	Tirzepatide 5, 10, or 15 mg SC once a week vs. placebo	Insulin glargine or insulin glargine + metformin
SURPASS-6 [57]	NCT04537923	Active, not recruiting	1182	HbA1c 7.5–11%, BMI ≥ 23 and ≤ 45 kg/m^2^ at screening Stable background medications (insulin glargine (U100) ± metformin)	eGFR < 30 ml/min/1.73m^2^ (< 45 if treated with metformin)	Tirzepatide 5, 10, or 15 mg SC once a week vs. insulin lispro (U100) SC three times a day with insulin glargine (U100) SC	Insulin glargine or insulin glargine + metformin

*SC* subcutaneous

**Table 5:** Primary (mean change in HbA1c from baseline at study end) and key secondary outcomes of the SURPASS 1–5 clinical trials with the obtained results

Study name	Primary/secondary endpoint(s) (weeks)	Mean HbA1c at baseline (%)	Mean HbA1c reduction from baseline (%) with tirzepatide 5/10/15 mg	Percentage of patients who met ^+^HbA1c < 7.0%; ^#^HbA1c ≤ 6.5% and ^*^HbA1c < 5.7% with tirzepatide 5/10/15 mg	Mean body weight at baseline (kg)	Mean reduction in body weight from baseline (kg) with tirzepatide 5/10/15 mg	Percentage of patients who met a weight loss of ^a^≥ 5%; ^b^≥ 10%; and ^c^≥ 15% with tirzepatide 5/10/15 mg
SURPASS-1 [38]	40	7.94	1.87/1.89/2.07	^+^87/92/88 ^#^82/81/86 *34/31/52	85.9	7.0/7.8/9.5	^a^67/78/77 ^b^31/40 /47 ^c^13/17/27
SURPASS-2 [39]	40	8.28	2.01/2.24/2.30	^+^82/86/86 ^#^69/77/80 ^*^27/40/46	93.7	7.6/9.3/11.2	^a^65/76/80 ^b^34/47/57 ^c^15/24/36
SURPASS-3 [40]	52	8.17	1.93/2.20/2.37	^+^82/90/93 ^#^71/80/85 ^*^26/39/48	94.3	7.5/10.7/12.9	^a^66/84/88 ^b^37/56/69 ^c^13/28/43
SURPASS-4 [41]	52	8.52	2.24/2.43/2.58	^+^81/88/91 ^#^66/76/81 ^*^23/33/43	90.3	7.1/9.5/11.7	^a^63/78/85 ^b^36/53/66 ^c^14/24/37
SURPASS-5 [42]	40	8.31	2.11/2.40/2.34	^+^87/90/85 ^#^74/86/80 ^*^24/42/50	95.2	5.4/7.5/8.8	^a^56/68/85 ^b^24/49/48 ^c^8/28/27

Results of the SURPASS-6 trial are not yet available

The SURPASS clinical trial program aims at evaluating the efficacy and safety of tirzepatide, a novel, once-weekly injectable dual glucose-dependent insulinotropic polypeptide (GIP) and GLP-1 receptor agonist (GIP/GLP-1 RA). The program includes seven global trials (SURPASS 1 to 6 and the SURPASS-CVOT), two trials for the Japanese market (SURPASS J-mono and SURPASS J-combo), and one trial for the Asia Pacific region (China) (SURPASS-AP-Combo) [56].

The SURPASS 1–6 randomized phase 3 trials aimed to evaluate the efficacy and safety of tirzepatide as a glucose-lowering medication in people with T2D. All patients in SURPASS 1–6 were ≥ 18 years, had T2D, and had a stable weight (± 5%) for at least 3 months. In case of background medication use, this had to be stable for at least 3 months before screening. Recruited patients had an HbA1c range ≥ 7.0% (SURPASS-6: ≥ 7.5%) and ≤ 10.5% (SURPASS-1: ≤ 9.5%; SURPASS-6: ≤ 11.0%). The body mass index (BMI) was ≥ 23 kg/m^2^ or ≥ 25 kg/m^2^ depending on the trial (Table 4). A further commonality was the random assignment of the participants to a once-weekly subcutaneous injection of tirzepatide (either 5 mg, 10 mg, or 15 mg). The starting dose (2.5 mg) was increased gradually at 4-week intervals to mitigate gastrointestinal side effects from the GLP-1 RA. Common key exclusion criteria were type 1 diabetes mellitus (T1D), history of pancreatitis, history of proliferative diabetic retinopathy, diabetic maculopathy, or non-proliferative diabetic retinopathy requiring acute treatment. The studies were conducted for 40–52 weeks.

The primary endpoint was the mean change in HbA1c from baseline at 40–52 weeks, and the key secondary endpoint was the mean reduction in body weight from baseline at 40–52 weeks. Additionally, the proportion of participants who reached the HbA1c target < 7.0%, ≤ 6.5%, and < 5.7%, and the percentage of those who achieved weight loss ≥ 5%, ≥ 10%, and ≥ 15% was evaluated. To date, the results of SURPASS 1–5 phase 3 trials have been published [38–42].

SURPASS-1 tested the effect of tirzepatide versus placebo in participants with an inadequately controlled HbA1c with diet and exercise alone. The tirzepatide-treated group showed a significant dose-dependent decrease in HbA1c from baseline by 1.87–2.07% versus an increase of 0.04% in the placebo group. 87% to 92% of the participants reached the target HbA1c < 7.0%. Regarding body weight, a significant weight loss of 7.0–9.5 kg compared to placebo (0.7 kg) could be observed. 67% to 78% of the participants reached a weight loss of 5% or greater with tirzepatide versus 14% with placebo (Table 5) [38].

In SURPASS-2, the efficacy and safety of tirzepatide versus semaglutide were investigated. The randomly assigned patients received a once-weekly subcutaneous injection of either tirzepatide or semaglutide (1 mg). In addition, all participants received metformin (≥ 1500 mg per day) [39]. With the three tested concentrations of tirzepatide, a significantly higher reduction of HbA1c and body weight from baseline could be achieved compared to semaglutide. Furthermore, significantly more participants reached the HbA1c targets < 7% and < 5.7% with 10 mg and 15 mg doses than with semaglutide. Further results are presented in Table 5 [39].

SURPASS-3 compared tirzepatide with insulin degludec in insulin-naive patients in whom oral glucose lowering drugs had failed to achieve therapeutic goals. The baseline therapy consisted of metformin with or without SGLT2is. The randomly assigned participants received either tirzepatide or once-daily insulin degludec (initially given at 10 Units per day and titrated once weekly to a fasting self-monitored blood glucose of less than 90 mg/dl) (Table 4) [40]. Independently of the doses, a significant reduction in HbA1c and body weight at week 52 could be observed with tirzepatide compared to insulin degludec. Additionally, significantly more patients achieved the HbA1c targets < 7.0%, ≤ 6.5%, and < 5.7 and the weight loss targets (≥ 5%, ≥ 10%, and ≥ 15% of body weight) (Table 5) [40].

In SURPASS-4, tirzepatide’s comparator was insulin glargine (100 U/ml). Participants in each group remained on currently prescribed treatment with metformin, SGLT2is, and/or sulfonylureas. All three doses of tirzepatide led to a significant reduction of HbA1c (− 2.24% (5 mg), − 2.43% (10 mg), and − 2.58% (15 mg) vs. − 1.44% insulin glargine) and body weight [− 7.1 kg (5 mg), − 9.5 kg (10 mg), and − 11.7 kg (15 mg) vs. + 1.9 kg (insulin glargine)] from baseline. Significantly more patients achieved the HbA1c targets < 7.0% and < 5.7%. No increase in adjusted MACE-4 events (CV death, MI, stroke, hospitalization for unstable angina) was noticeable on tirzepatide compared with insulin glargine (HR 0.74 [95% CI 0.51–1.08]). Further results are shown in Table 5 [41].

In SURPASS-5, tirzepatide was compared with placebo. Participants received insulin glargine (U100), once daily with or without metformin as background medication (Table 4). Tirzepatide 5 mg, 10 mg, and 15 mg was superior to placebo in HbA1c change from baseline, body weight reduction, and percentage of participants achieving glycemic and weight loss targets (Table 5) [42].

The most common adverse effects in all SURPASS 1–5 trials of the tirzepatide-treated groups were gastrointestinal (mainly nausea, vomiting, and diarrhea). Their severity was generally mild to moderate and mainly dose-dependent. They occurred more frequently in the tirzepatide-treated groups than in the comparator groups. The incidence of severe hypoglycemia and blood glucose concentrations less than 54 mg/dl was lower with tirzepatide compared to insulin degludec and insulin glargine respectively (SURPASS-3 and 4) or did not differ between the tirzepatide and the comparator groups (SURPASS-1, 2, and 5) [38–42].

The SURPASS-CVOT, whose estimated completion date is October 2024, aims to investigate the cardiovascular safety of tirzepatide compared to dulaglutide 1.5 mg. The primary endpoint is a composite of MI, stroke, and CV death over 52 weeks. In SURMOUNT-1 the effect of tirzepatide primarily on weight loss in people with diabetes and obesity is being investigated. Primary outcomes are body reduction from baseline and the percentage of participants achieving ≥ 5% body weight reduction by week 72.

## Obesity and overweight outcome trials

### STEP trials: (Table 6)

**Table 6:** Key information of the STEP 1–5 clinical trials with the GLP-1 receptor agonist semaglutide 2.4 mg

Study name	Clinicaltrials.gov ID	Study status	n	Participants	Intervention	Primary outcome	Mean body weight at baseline (kg)	Mean percentage change in body weight from baseline semaglutide 2.4 mg vs. placebo	Percentage of patients who met a weight loss of ≥ 5% with semaglutide 2.4 mg vs. placebo
STEP 1 [43]	NCT03548935	Completed	1961	With obesity or overweight, without T2D	Semaglutide 2.4 mg once a week vs. placebo	Percentage change in body weight and weight reduction of at least 5% at week 68	105.3	− 14.9% vs. − 2.4%	86.4% vs. 31.5%
STEP 2 [44]	NCT03552757	Completed	1210	With obesity or overweight, with T2D	Semaglutide 2.4 mg once a week vs. semaglutide 1.0 mg and placebo	Percentage change in body weight and weight reduction of at least 5% at week 68	99.8	− 9.6% vs. − 3.4%	68.8% vs. 28.5%
STEP 3 [45]	NCT03611582	Completed	611	With obesity or overweight, without T2D	Semaglutide 2.4 mg once a week vs. placebo in addition to intensive behavioral therapy	Percentage change in body weight and weight reduction of at least 5% at week 68	105.8	− 16.0% vs. − 5.7%	86.6% vs. 47.6%
STEP 4 [46]	NCT03548987	Completed	902	With obesity or overweight, without T2D	Semaglutide 2.4 mg once a week for the first 20 weeks, then random assignment: semaglutide 2.4 mg once a week vs. placebo for 48 weeks	Percent change in body weight from week 20 to week 68	107.2	− 7.9% vs. + 6.9%	Not applicable
STEP 5 [58]	NCT03693430	Completed	304	With obesity or overweight, without T2D	Semaglutide 2.4 mg once a week vs. placebo	Percentage change in body weight and weight reduction of at least 5% at week 104	106.0	− 15.2% vs. − 2.6%	77.1% vs. 34.4

The STEP trial 1 to 5 investigated the effect of semaglutide 2.4 mg as adjunctive therapy to lifestyle intervention on weight loss in adults with obesity or overweight [43–46, 58].

In these randomized, double-blind trials, ≥ 18 years old participants without T2D and with a BMI ≥ 30 kg/m^2^ or ≥ 27 kg/m^2^ and one or more treated or untreated weight-related comorbidities (hypertension, dyslipidemia, obstructive sleep apnea, or CV disease) were included. An exception was the STEP 2 trial, which explicitly included patients with T2D and BMI ≥ 30 kg/m^2^ or ≥ 27 kg/m^2^.

The primary endpoints were the percentage change in body weight from baseline to the end of treatment (STEP 4: from week 20 to week 68) and the proportion of participants achieving ≥ 5% weight loss from baseline after the end of treatment (except for STEP 4) (Table 6) [43–46, 58].

In the STEP 1 trial, semaglutide 2.4 mg was associated with a significant reduction in body weight (− 14.9%) compared to placebo (− 2.4%). Moreover, significantly more participants achieved a weight loss ≥ 5% with semaglutide than with placebo (86.4% vs. 31.5%).

In STEP 2, which included participants with T2D, the percentage change in mean body weight was − 9.6% with semaglutide 2.4 mg and − 3.4% with placebo. At week 68, significantly more participants achieved a weight loss of ≥ 5% with semaglutide than with placebo (68.8% vs. 28.5%).

In the STEP 3 trial, the lifestyle intervention was more intensive than STEP 1 (low-/hypocaloric diet, weekly physical activity, and behavioral therapy versus behavioral counselling visits every 4 weeks in STEP 1). The mean body weight change from baseline was 16.0% for semaglutide 2.4 mg and 5.7% for placebo. Compared with placebo, significantly more participants treated with semaglutide lost ≥ 5% body weight.

In STEP 4, all the participants received semaglutide 2.4 mg for 20 weeks (run-in period). They were then randomized to continue the semaglutide treatment or to switch to placebo for 48 weeks. The weight loss effect of continuing semaglutide treatment versus switching to placebo from week 20 to week 68 was investigated. After completion of the run-in period, participants achieved a mean weight loss of 10.6%. The mean body change from week 20 to week 68 was − 7.9% for semaglutide and + 6.9% for placebo, indicating a maintained weight loss effect for the treatment with semaglutide 2.4 mg [46].

In the STEP 5 trial, the long-term weight loss effect of semaglutide was evaluated [semaglutide 2.4 mg vs. placebo for 2 years (104 weeks)]. The participants treated with semaglutide had, on average, a significant and sustained weight loss compared to those treated with placebo (15.2% vs. 2.6%) and were more likely to achieve a weight loss ≥ 5% (Table 6) [58].

The most reported adverse events in the STEP 1–5 trials were nausea and diarrhea. These were mild to moderate and were more frequent with semaglutide 2.4 mg than placebo [43–46, 58].

## Key topics discussed during the 7th CVOT Summit

### Key aspects of the ESC heart failure guidelines 2021

In the 2021 European Society of Cardiology (ESC) Guidelines for the diagnosis and treatment of acute and chronic heart failure [59], the definition of HF with mildly reduced ejection fraction (HFmrEF) was slightly modified. A mildly reduced ejection fraction (HFmrEF) is defined as a range of 41–49%. Elevated natriuretic peptides, structural heart disease, or diastolic dysfunction are no longer required criteria. Regarding recommendations for pharmacological treatments of HF with reduced ejection fraction (HFrEF; LVEF ≤ 40%), the guidelines issued a class I; level A (IA) recommendation for angiotensin-converting-enzyme inhibitors (ACEis), beta-blockers, MRAs, and the SGLT2is dapagliflozin and empagliflozin to reduce the risk of HHF and death [59]. Angiotensin-receptor blockers (ARBs) are recommended (class I, level B) to reduce the risk of HHF and CV death in symptomatic patients unable to tolerate ACEis or angiotensin receptor-neprilysin inhibitors (ARNIs). Loop diuretics are still recommended for patients with congestion symptoms (class I, level C). The therapeutic algorithm for the management of patients with HFrEF has been updated accordingly. It is now recommended to initiate therapy with ACEis/ARBs, Beta-blockers, MRAs, and dapagliflozin/empagliflozin simultaneously; loop diuretics are prescribed for fluid retention. The SGLT2is canagliflozin, dapagliflozin, empagliflozin, ertugliflozin and sotagliflozin received the recommendation class I for persons with T2D at risk for CV events. For persons with T2D and HFrEF, dapagliflozin, empagliflozin, and sotagliflozin were recommended (class I). The guidelines emphasize patient profiling and phenotyping to personalize medication use and achieve the best possible therapeutic effect [59, 60].

### The “living guidelines” approach—the future of guideline creation?

More than 660,000 publications and 5300 registered or ongoing trials related to COVID-19 have been recorded. In the light of constantly and rapidly emerging new research evidence, the publication of systematic reviews including the latest evidence can become a complicated and resource-demanding task that affects the timely update of guidelines. The “living guidelines” approach, meaning the continuous update of the guideline recommendations and sustained by advances in evidence-based medicine and digitalization technology, was developed to tackle this challenge and create trustworthy, timely, and accessible guidelines [61, 62]. The core of living guidelines are high-quality, accessible systematic reviews that are constantly kept updated (living systematic reviews) and potentially network meta-analyses [63]. Regarding living guidelines for COVID-19, the WHO, the Australian National COVID-19 Clinical Evidence Taskforce, the National Institute for Health and Care Excellence (NICE) in the UK, and the Association of the Scientific Medical Societies in Germany (AWMF) in cooperation with COVID-19 evidence ecosystem (CEOsys) used the web-based platform MAGICapp of the nonprofit organization MAGIC Evidence Ecosystem Foundation to develop and disseminate COVID-19 living guidelines [64–69]. Concerning diabetes, the Australian Living Evidence for Diabetes Consortium has already published living guidelines preceding the COVID-19 breakthrough [62, 70].

In 2019, an interdisciplinary experts’ panel (Taskforce of the Guideline Workshop) convened to develop strategies to optimize guideline processes in diabetes, CVD, and kidney diseases [71]. In 2020 The Taskforce initiated a pilot project supporting the creation of evidence-based guidelines for the use of SGLT2is and GLP-1 RAs to manage very high risk T2D patients (presence of both CVD and DKD) using the MAGICapp platform [72]. Importantly, this guideline was based on a high-quality systematic review and network meta-analysis of these drugs in T2D patients, demonstrating moderate to high certainty evidence for their beneficial effects on cardiorenal outcomes [73].

One of the Taskforce’s conclusions from this successful pilot was to move towards living guidelines for cardiorenal outcomes in diabetes, as demonstrated in the COVID-19 pandemic. The Taskforce will now be included in an update of the above-mentioned systematic review and network meta-analysis. The addition of new CVOT trials, and in particular determining the relative effectiveness of finerenone, can help the societies update their respective guidelines. The next goal would be for the professional societies to move to living guidelines, based on living systematic reviews with network meta-analysis to inform dynamic and rapid updates of recommendations.

### Kidney disease due to diabetes—FIDELITY meta-analysis

FIDELITY was designed as a prespecified individual meta-analysis of the trials FIDELIO-DKD and FIGARO-DKD (data of 13.026 persons with T2D and CKD; median follow up of 3 years) [34, 36, 74]. The CV outcome was a composite of time to first occurrence of CV death, non-fatal MI, non-fatal stroke, or HHF. The kidney outcome was a composite of time to first occurrence of kidney failure, a sustained ≥ 57% decrease in eGFR from baseline over 4 weeks, or renal death. The composite CV outcome occurred in 12.7% of the finerenone-treated group and 14.4% of the placebo-treated group (HR 0.86 [95% CI 0.78–0.95]; p = 0.0018). The relative risk of the composite kidney outcome was significantly reduced by 23% with finerenone (occurrence of a kidney-related event in 5.5% of the individuals who received finerenone and 7.1% of those treated with a placebo (HR, 0.77 [95% CI 0.67–0.88]; p = 0.0002). Hyperkalemia leading to permanent treatment discontinuation was more frequent in the finerenone-treated group than in the placebo group (1.7% vs. 0.6%) [74].

### DARE-19 trial

Since COVID-19 may impair multiple organs, through among others vascular damage, endothelial dysfunction, and inflammation resulting in thrombosis and potential organ damage [75] and knowing the significant protective effects of dapagliflozin on the heart and kidney [25, 26], the Dapagliflozin in Respiratory failure in patients with COVID-19 trial (DARE-19) was conducted to evaluate the organ-protective effect of dapagliflozin in patients with cardiometabolic risk factors hospitalized with COVID-19 [76]. The trial included patients hospitalized for COVID-19 with at least one cardiometabolic risk factor (i.e., atherosclerotic CVD, hypertension, T2D, HF, and CKD). Key exclusion criteria were critical illness, eGFR < 25 ml/min/1.73m^2^, T1D, and prior diabetic ketoacidosis. The participants were randomized to receive dapagliflozin (10 mg/day) or a placebo for 30 days. The trial had dual composite primary endpoints: a prevention endpoint (time to new or worsened organ dysfunction or death from any cause) and a hierarchical recovery endpoint (change in clinical status by day 30). Safety outcomes (in patients who received ≥ 1 study medication dose) included serious adverse events, adverse events leading to discontinuation, and adverse events of interest. The prevention endpoint (time to organ dysfunction or death) occurred in 70 patients (11.2%) in the dapagliflozin group, and 86 (13.8%) in the placebo group (HR 0.80 [95% CI 0.58–1.10]; p = 0.17). The primary outcome of recovery (clinical status improvement) was numerically in favor of the dapagliflozin group than the placebo group [n = 547 (87.5%) vs. n = 532 (85.1%)]; however, this was statistically not significant (win ratio 1.09 [95% CI 0.97–1.22]; p = 0.14). Serious adverse events occurred in 65 (10.6%) of 613 dapagliflozin-treated participants and in 82 (13.3%) of 616 patients who received a placebo [76]. Although some recommendations suggest stopping SGLT2is in case of a COVID-19 infection in people with diabetes [77, 78], the results provided by the DARE-19 trial do not support the discontinuation of SGLT2is as long as patients with COVID-19 and cardiometabolic risk factors are monitored. The DARE-19 results have already led to an update of consensus recommendations on COVID-19 and metabolic disease [79].

### Insulins and the glycemic management

#### Biosimilar insulins

The World Health Organization (WHO), the FDA, and the European Medicines Agency (EMA) define biosimilars as biotherapeutic/biological products/medicines that are highly similar to already approved biotherapeutic/biological products/medicines [80–82]. Biosimilar insulins are intended to have the same effect in the human body, at the same dose level, and therefore should be taken in the same way as the original (reference) insulin. Although biosimilar insulins are manufactured using the same human genome sequence as the reference insulin, they cannot be exact copies of the reference insulin due to differences in the manufacturing process of biologics [83, 84]. Minor differences in clinical action may exist, but a biosimilar product only receives regulatory approval after demonstrating its high similarity to the reference product with no meaningful differences in terms of safety, purity, and potency based on its “totality of evidence” [85]. This stepwise approach to establish biosimilarity includes comparative assessments, preclinical cell-based and animal studies, and clinical studies in humans. Deviation at the end-stage, including receptor binding, pharmacokinetic and pharmacodynamic studies, and immunogenicity profile, have a critical impact on regulatory decisions [86]. As adopted in Europe, the USA, and many other strictly regulated countries, these requirements are designed to prevent products of substandard quality from entering the market [87, 88]. Because of the typically shorter development period, biosimilars provide a more cost-effective treatment option [89]. This ensures stronger competitiveness and can improve the affordability and accessibility of persons with diabetes to appropriate insulin therapy.

#### Perspectives of the insulin therapy

The prospective trial ORIGIN, which involves people with CV risk factors and impaired fasting glucose, impaired glucose tolerance, or T2D, showed a neutral effect of insulin glargine on CV outcomes (risk for nonfatal MI, nonfatal stroke, or death from CV causes and these ± revascularization or HHF) and cancer compared to standard-care [90]. In addition, the DEVOTE trial showed no difference in the risk of 3-point MACE (CV death, nonfatal MI, or nonfatal stroke) between insulin degludec and insulin glargine U100 in persons with T2D at high risk for CV events [91]. The results of the completed GRADE trial comparing insulin glargine, glimepiride, sitagliptin, and liraglutide in combination with metformin regarding their efficacy and, among others, CVD risk factors have yet to be published, but preliminary results indicate comparable effects of insulin glargine, glimepiride, and sitagliptin concerning the combined CVD endpoint [92, 93].

#### Glycemic management

Major clinical trials conducted in the last decades regarding diabetes management used the surrogate, long-term marker HbA1c to assess the efficacy of diabetes care in routine clinical care for both T1D and T2D [94, 95]. Improvements in HbA1c levels significantly reduced the risk of microvascular complications [94, 95]. However, in some T2D trials, a tight HbA1c-guided metabolic control led to increased overall mortality, possibly due to a higher rate of hypoglycemic events in the intensive treatment arm [96–98]. By its physiological nature, the HbA1c has some limitations. It does not reflect glycemic variability or hypo- and hypoglycemic excursions [99]. Besides, various factors (e.g., hemoglobinopathies, CKD, individual changes in red blood cells lifespan) may lead to interindividual glycation variabilities affecting the accuracy and informative value of HbA1c [100, 101]. Parallel to HbA1c, continuous glucose monitoring (CGM) has seen a strong development over the past decade. The improvements were not only reflected in the development of better algorithms and more accurate interstitial sensors [102–104], but also in the standardization of CGM metrics and their clear visualization using a single-page report (Ambulatory Glucose Profile (AGP) report) [105]. Although no results of long-term studies using CGM are available to date, an analysis using data of the seven-point blood glucose found an association of the time in range (TIR) (70–180 mg/dl) and the progression of retinopathy and microalbuminuria in T1D. The retinopathy progression and microalbuminuria outcome increased significantly with lesser TIR [106]. Also, in T2D, a negative correlation between TIR and both diabetic retinopathy and neuropathy was detected [107, 108]. A more recent prospective cohort study with 6225 patients with T2D using CGM indicated that a lower TIR correlated with an increased risk of all-cause and CV mortality [109]. It is also to mention that, in contrast to HbA1c, CGM metrics allow the assessment of glucose variability and hypo- and hyperglycemic excursions [105]. Regarding regulatory measures, the American Diabetes Association (ADA), in its Standard of Medical Care in Diabetes, 2021 referred, that glycemic control is assessable by HbA1c measurement, CGM, and self-monitoring of blood glucose (SMBG) and attributed CGM an essential role in both T1D and T2D [110]. In 2018 the FDA approved the first CGM system with a fully implantable glucose sensor [111] and, in 2020, expanded the availability and capability of non-invasive remote monitoring devices, including CGM systems, because of the COVID-19 pandemic [112]. This evolution indicates the growing importance of CGM in diabetes management alongside HbA1c.

## Conclusion

The 7th Cardiovascular Outcome Trial (CVOT) Summit on Cardiovascular, Renal and Glycemic Outcomes offered an interactive and multi-disciplinary platform to discuss key results of recently published trials. The virtual format enabled attendants from 88 countries to participate. The summit covered two CVOTs (FIGARO-DKD and AMPLITUDE-O) and one HF trial (EMPEROR-Preserved). In addition, glycemic (SURPASS 1–5) and overweight/obesity outcome trials (STEP 1–5) were discussed. The meeting provided novel data, insights, strategies, and guidelines for specialists and primary care for the management of diabetes, obesity, HF, CV, and kidney disease. In-depth discussions and presentations of upcoming CV, kidney, HF, glycemic, and obesity trials will be resumed at the 8th edition of the CVOT Summit, which will be held virtually on November 10–11, 2022 (https://www.cvot.org).

## Data Availability

Data sharing not applicable to this article as no datasets were generated during the current study.

## Abbreviations

ACEi: Angiotensin-converting-enzyme inhibitor
ADA: American Diabetes Association
AGP: Ambulatory Glucose Profile
ARB: Angiotensin-receptor blocker
ARNI: Angiotensin receptor-neprilysin inhibitor
AWMF: Arbeitsgemeinschaft der Wissenschaftlichen Medizinischen Fachgesellschaften
BMI: Body mass index
CGM: Continuous glucose monitoring
CI: Confidence interval
CKD: Chronic kidney disease
CV: Cardiovascular
CVD: Cardiovascular disease
CVOT: Cardiovascular outcome trial
DKD: Kidney disease due to diabetes
DPP-4i: Dipeptidylpeptidase-4 inhibitor
eGFR: Estimated glomerular filtration rate
EMA: European Medicines Agency
ESC: European Society of Cardiology
FDA: U.S. Food and Drug Administration
GIP: Glucose-dependent insulinotropic polypeptide
GLP-1 RA: Glucagon-like peptide-1 receptor agonist
HbA1c: Glycated hemoglobin 1Ac
HF: Heart failure
HFmrEF: Heart failure with mildly reduced ejection fraction
HFpEF: Heart failure with preserved ejection fraction
HFrEF: Heart failure with reserved ejection fraction
HHF: Hospitalization for heart failure
HR: Hazard ratio
IDF: International diabetes federation
KCCQ: Kansas City Cardiomyopathy Questionnaire
LVEF: Left ventricular ejection fraction
MACE: Major adverse cardiovascular event
MI: Myocardial infarction
MRA: Mineralocorticoid receptor antagonist
NICE: National Institute for Health and Care Excellence
NYHA: New York Heart Association
RAS: Renin-angiotensin system
SC: Subcutaneous
SGLT2i: Sodium–glucose cotransporter-2 inhibitor
SMBG: Self-monitoring of blood glucose
T1D: Type 1 diabetes mellitus
T2D: Type 2 diabetes mellitus
TIR: Time in Range
UACR: Urine albumin-to-creatinine ratio
WHO: World Health Organization
